# Delirium in nursing homes and long-term care facilities: findings of a scoping review of detection tools

**DOI:** 10.1007/s41999-025-01250-8

**Published:** 2025-06-28

**Authors:** Irene Mansutti, Chiara Muzzana, Vanessa Vater, Pia Urfer Dettwiler, Alvisa Palese, Dietmar Ausserhofer, Wolfgang Hasemann

**Affiliations:** 1 Nursing Science, Department of Medicine, University of Udine, Udine, Italy; 2 Claudiana Research, College of Healthcare-Professions Claudiana, Bolzano-Bozen, Italy; 3 Nursing Research, University Hospital of Frankfurt, Frankfurt, Germany; 4 Institute of Nursing Science, University of Basel, Basel, Switzerland; 5 Medizinische Fakultät|Department of Public Health (DPH), Institut Für Pflegewissenschaft-Nursing Science (INS), Universität Basel, Bernoullistrasse 28, 4056 Basel, Schweiz

**Keywords:** Delirium, Diagnosis, Diagnostic techniques and procedures, Psychometrics, Tools, Instruments, Validation, Nursing homes, Long-term care, Neurocognitive disorders

## Abstract

**Background:**

The detection of delirium among residents in nursing homes (NH) and long-term care (LTC) facilities remains challenging due to overlapping symptoms with dementia, limited staff training, and variability in assessment tools. This scoping review aimed to identify delirium detection tools and their properties as used for research purposes in these settings.

**Methods:**

The methodology was guided by Arksey and O’Malley and Levac et al. Medline, Embase, CINAHL, PsycINFO, Cochrane Database of Systematic Reviews, and Cochrane Central Register of Controlled Trials were searched for original research studies.

**Results:**

A total of 58 studies met the inclusion criteria, with most conducted in the USA (37.9%) and Europe. We identified 25 delirium detection tools, with the Confusion Assessment Method long form (43.1%), the Neelon and Champagne Confusion Scale (10.3%), the Nursing Home Confusion Assessment Method (10.3%), the short Confusion Assessment Method and its modifications (8.6%), the Delirium Index (6.8%), the Delirium Rating Scale-Revised-98 (6.8%), and the 4A’s Test (5.1%) as the most frequently used. Only 14 tools have undergone validity and reliability testing in NH/LTC settings, with the Delirium Observation Screening Scale showing the highest diagnostic accuracy. Ten tools showed full concordance regarding the three delirium domains: cognitive domain, higher-level thinking domain, and circadian domain. The Delirium Diagnostic Tool-Provisional (DDT-Pro) required no more than three items.

**Discussion:**

This review highlights the lack of standardized delirium detection in NH/LTC settings. Future research should focus on providing evidence based on validity and reliability of existing tools, integrating delirium screening and assessment into routine care as part of delirium prevention and management, and training NH/LTC staff.

**Supplementary Information:**

The online version contains supplementary material available at 10.1007/s41999-025-01250-8.

## Key summary points

**Aim:**

To identify and evaluate delirium detection tools and their psychometric properties in nursing homes and long-term care (LTC) settings.

**Findings:**

A total of 25 delirium detection tools were identified, with the Confusion Assessment Method (CAM) and its variants being the most frequently used. Only 14 tools have undergone validity and reliability testing in LTC settings, with the Delirium Observation Screening Scale (DOSS) showing the highest diagnostic accuracy. The Delirium Diagnostic Tool-Provisional (DDT-Pro) requires the least number of items to cover all three delirium domains.

**Message:**

There is a need for standardized delirium detection tools and improved staff training in LTC settings to enhance early detection and management of delirium.

**Supplementary Information:**

The online version contains supplementary material available at 10.1007/s41999-025-01250-8.

## Background

Delirium is a neuropsychiatric disorder that develops in the context of acute illness [1, 2]. Its prevalence in NH/LTC ranges from 8.9 to 37.8%, depending on the measurement tool and country [3–9]. It is considered a medical emergency, because failure to address the underlying cause can lead to long-term consequences, including increased frailty, accelerated progression of dementia and higher mortality [10–12]. Delirium is associated with increased workload for health care workers and the emotional distress of residents and their families [13, 14].

A common reason for the lack of treatment is the frequent under or non-detection of the condition [15, 16]. This diagnostic gap is particularly pronounced in LTC settings, due to a combination of (1) resident demographics, (2) staffing structures, (3) the status of delirium prevention and management and (4) the level of advancement in internal policies, thus, influencing how delirium is detected and who is responsible for its identification in these facilities. Given the demographic profile of residents, delirium exhibits overlapping features with other neurocognitive disorders such as dementia [17, 18]. Consequently, the coexistence of both conditions must be considered, which complicates the detection and differentiation of delirium in this population [19]. Staffing structures, i.e., nursing skill and grade mix differ from that of hospitals, featuring a higher proportion of less-qualified nursing assistants and fewer registered nurses [20]. Advanced practice nurses, who have achieved a master’s degree, are an exception in LTC facilities [21, 22]. The near absence of national guidelines for delirium prevention and management in LTC settings, further hampers the recognition of delirium [23].

Over the past five decades, approximately 89 tools have been developed to assess delirium [24–26], beginning with Lowy’s Delirium Scale (D-Scale) in 1973 [27]. Most delirium detection tools were designed for use in adult hospital settings, with only a limited number validated for application in other settings [28, 29]. However, it remains unclear which of these tools are appropriate for use in NH and LTC settings. Little is known about the adaptation/modification, validity and reliability and users (e.g., research and/or clinical staff) of these tools in NH and LTC settings [13]. Thus, due to differences in resident profiles and staffing structures, tools validated in acute hospital settings may not perform reliably in LTC environments.

With this article we aim to provide a comprehensive overview of delirium detection tools used in NH and LTC settings, summarize the validity and reliability of these tools, and provide future directions for clinical practice and research on detecting delirium in older adults living in NH/LTC settings.

## Materials and methods

### Design

This scoping review was guided by the methodological framework of Arksey and O’Malley [30] and Levac et al. [31] and performed in accordance with the Preferred Reporting Items for Systematic Review and Meta-analysis extension for Scoping Reviews (PRISMA–ScR) [32].

### Data sources and search strategy

PubMed–Medline, Embase, CINAHL, PsycInfo, Cochrane Database of Systematic Reviews and Cochrane Central Register of Controlled Trials were searched in December 2024 using MeSH/Thesaurus and free terms (e.g., delirium, acute confusion, long-term care, nursing homes, nursing skilled facilities, tool, and screening). An overview of the search strategy is presented in Supplementary Appendix S1. A time limitation was set to studies published after 2000, as this was the publication year of DSM-IV-TR [33].

### Study selection

**Fig. 1:**
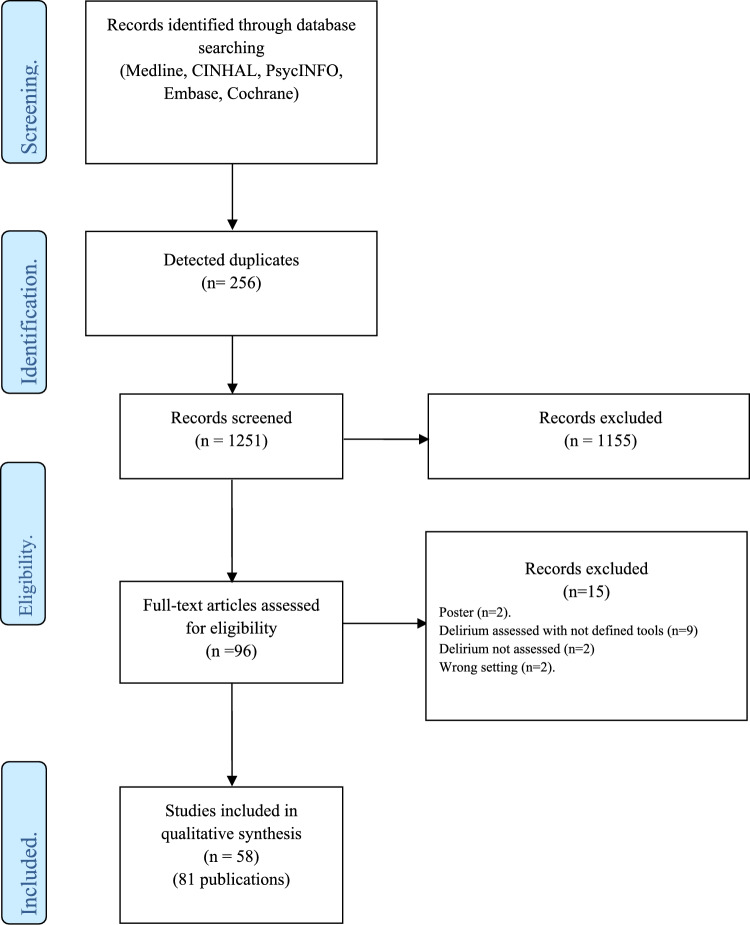
PRISMA flowchart

Figure 1 shows the process of study selection and inclusion. Four independent reviewers (CM, DA, IM, VV) screened the titles and abstracts to identify relevant records. Differences were discussed with a fifth researcher (PUD). Each record was screened by two reviewers according to the following inclusion/exclusion criteria:

1. Inclusion criteria: (i) original research articles reporting empirical data; (ii) performed in NH or LTC facilities (hereinafter NHs); (iii) evaluating the presence of delirium; (iv) reporting or describing tools or a classification system used to assess delirium (with or without psychometric properties reported); (v) published in English, German or Italian languages, based on the language competencies of the review team.
2. Exclusion criteria: (i) qualitative studies, letters to the editors, commentaries, study protocols, or congress posters; (ii) focused on hospital, indwelling or home care settings, or on pediatric populations; (iii) assessing only cognitive impairment, dementia and/or other psychiatric diseases or symptoms (e.g., depression, agitation, aggression, and behavioral issues), but not delirium and (iv) not using a structured assessment/screening tool.

Three literature reviews were analyzed for the included studies, to consider all the available original research articles on this topic [2, 13, 34].

### Data extraction

Seven reviewers (AP, CM, DA, IM, PUD, VV, WH) extracted the data from eligible studies into a spreadsheet for the following variables: first author, publication year, study design, number of participants, enrolment period, country and setting, delirium screening tools used, who performed the screening and, if available, evidence resulting from validity and reliability testing. The Supplementary Appendix S2 provides a comprehensive overview of the data extracted from the included publications and studies.

### Data analysis

According to the aims of the study, we first checked for each publication—based on the information reported in the method section (i.e., design, sample and setting, and delirium screening tools used)—if one study had resulted in more than one publication. Consequently, we summarized findings from all publications belonging to the same study. We analyzed a total of 82 relevant publications summarized to 58 studies (see Supplementary Appendix S2). Moreover, findings have been summarized narratively, and, regarding the psychometric properties, these were extracted as reported in the studies. Items of the delirium tools were aligned to the three delirium domains as described by Trzepacz et al. [35–40]

## Results

### Information on the included studies

Of the 58 included studies, 22 were conducted in the USA, followed by Canada and several European countries. The smallest study enrolled 22 NH residents [41], while the largest study analyzed data from 5′588′702 post-acute care patients in NH [42] (Table 1).

**Table 1:** General information on the studies included (*N* = 58)

	*n* (%)
Country	
USA	22 (37.9)
Canada	10 (17.2)
Spain	6 (10.3)
The Netherlands	1 (1.7)
Belgium	1 (1.7)
Italy	5 (8.6)
Switzerland	2 (3.4)
Norway	1 (1.7)
Finland and Sweden	3 (5.1)
England	3 (5.1)
Australia	1 (1.7)
Korea	1 (1.7)
Brazil	1 (1.7)
Israel	1 (1.7)
Years of publication	
2000–2010	15 (25.8)
2011–2020	34 (58.6)
2021–2024	9 (15.5)
Study design	
RCT	8 (13.7)
Quasi experimental	3 (5.1)
Cross section	13 (22.4)
Observational prospective	24 (41.3)
Observational retrospective	5 (8.6)
Case series	1 (1.7)
Secondary analysis	4 (6.8)
Sample size	
Median (quartiles 1st–3rd)	267.5 (140.5–769.75)
Sample mean age	
≥64 years	33 (56.9)
Not reported for the whole sample	25 (43.1)

*RCT* Randomized Controlled Trial

### Delirium detection tools and criteria

**Fig. 2:**
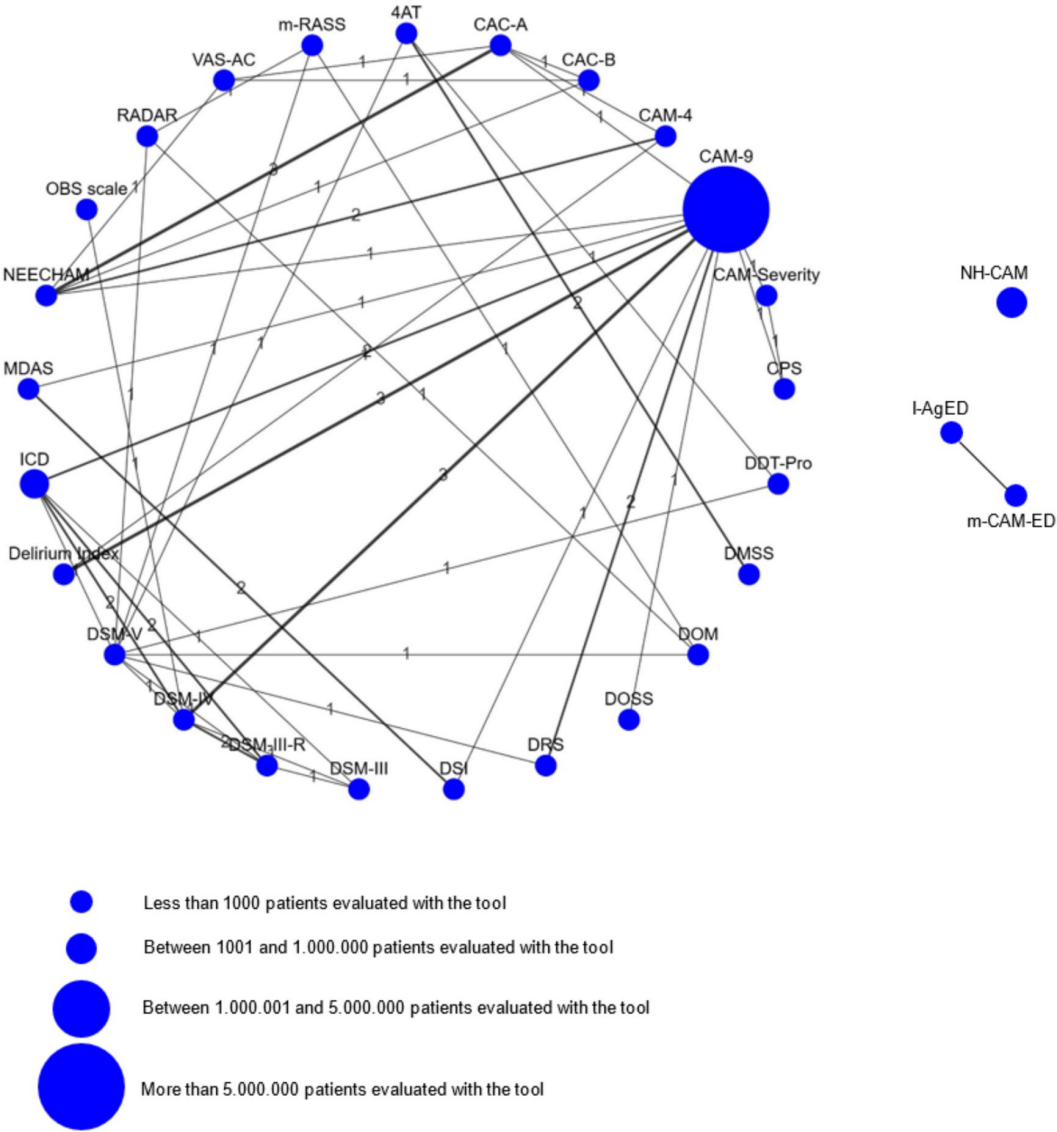
Delirium screening tools used and comparisons (the node size shows the total number of participants screened with a specific tool; the edge size shows how many studies used simultaneously more tools)

As described in Table 2, the most documented tools were the Confusion Assessment Method (CAM) [43]; the Neelon and Champagne Confusion Scale (NEECHAM) [44] and the Nursing Home Confusion Assessment method (NH-CAM) [45]. The long CAM [46] was prevalently used between 2006 and 2012, while the short CAM [43] or modified CAMs [47] were most used after 2013. The diagnostic criteria of the DSM in their editions [1, 48–50] were the most used ones for assessing delirium. In 22 studies the assessment was performed by researchers or research staff [4, 12, 51–70], and three studies reported the assessments were performed both by staff (nurses, physicians, geriatricians) and research team [3, 5, 71]. Ten studies did not report who performed the assessment [29, 45, 72–80] but most of them were conducted over 10 years ago. In 55 studies residents were directly screened or assessed for delirium, while in 4 studies [71, 81–83] data were used from the Minimum Data Set of the Resident Assessment Instrument or other data sources [84, 85]. As Fig. 2 describes, most studies (*n* = 36) used a combination of different tools and classification systems (DSM, ICD). More specifically, 29 studies (50%) detected delirium using only one tool, 19 studies (32.8%) used two tools, while 5 (8.6%), 4 (6.9%) and 1 (1.7%) used, respectively, three, four and five tools simultaneously. Frequently, in studies using more tools, one of them was a version of the DSM or the ICD (frequently considered as reference standards). The most frequently used tool was the CAM [43] (9 item version; 43.1% of studies), with 7′603′451 patients cumulatively evaluated among studies. The less used tools were the Delirium-O-Meter (DOM) [86] and the Recognizing Acute Delirium as Part of Your Routine (RADAR) Tool [87] (used to evaluate the presence of delirium in 22 patients, in the same study) [41].

**Table 2:** Criteria and delirium detection tools used in the NH/LTC based on the 58 included studies

Delirium tools and criteria	*n* (%)
CAM 9 or long OR unspecified [43]	25 (43.1)
DSM-IV[33]	7 (12.0) †
DSM-5 [50]	6 (10.3) †
NEECHAM [44]	6 (10.3) ‡
NH CAM [45]	6(10.3)
CAM (short, 4 item) [43]	5 (8.6)
ICD [88]	5 (8.6) †
Delirium Index [89]	4 (6.8)
DRS-R-98 [90]	4 (6.8)
DSM-III-R [49]	4 (6.8) †
RAI–MDS 3.0 [85]	3 (5.1)
4AT [40]	3 (5.1) ‡
CAC-A [91]	3 (5.1) ‡
CAM–S [92]	3 (5.1)
DMSS [93]	2 (3.4)
DSI [94]	2 (3.4)
MDAS [95]	2 (3.4)
CAC-B [96]	1 (1.7) ‡
DDT-Pro [40]	1 (1.7) ‡
DOSS 25 [97]	1 (1.7)
DOSS 13 [98]	1 (1.7) ‡
DOM [86]	1 (1.7)
DSM-III [48]	1 (1.7) †
IAGe-D [28]	1 (1.7) ‡
mCAM–ED [47]	1 (1.7)
OBS scale [99]	1 (1.7)
RADAR [87]	1 (1.7) ‡
VAS–AC [44, 100]	1(1.7) ‡
Raters	
Nursing staff and/or nurse assistants	9 (15.1)
Physicians, Psychiatrist, Geriatricians	6 (10.3)
Research nurses	3(5.1)
Research team	5 (8.6)
Not reported	10 (17.2)

*4AT* 4 ‘A’s Test (Rapid Assessment Test for Delirium), *CAC-B* Cognitive Assessment of Confusion—Behavioral, *CAC-A* Cognitive Assessment of Confusion—Acute, *CAM—S* Confusion Assessment Method—Severity, *CAM* Confusion Assessment Method, *DDT-Pro* Delirium Diagnostic Tool-Provisional, *Delirium Index* Delirium Index, *DMSS* Delirium Motor Subtype Scale, *DOM* Delirium-O-Meter, *DOSS* Delirium Observation Screening Scale, *DRS-R-98* Delirium Rating Scale-Revised-98, *DSI* Delirium Symptom Interview, *DSM-III* Diagnostic and Statistical Manual of Mental Disorders = Third Edition, *I-AGeD* Informant Assessment of Geriatric Delirium, *ICD* International Classification of Diseases, *mCAM–ED* modified Confusion Assessment Method for the Emergency Department, *MDAS* Memorial Delirium Assessment Scale, *NEECHAM* Neelon and Champagne Confusion Scale, *NH CAM* Nursing Home Confusion Assessment Method, *OBS scale* Organic Brain Syndrome Scale, *RADAR* Recognizing Acute Delirium as Part of Your Routine, *RAI–MDS 3.0* Resident Assessment Instrument–Minimum Data Set 3.0, *VAS–AC* Visual Analog Scale for Acute Confusion

^†^Classification system

^‡^ Evidence on validity and reliability in LTC/NH setting reported

### Depiction of the three domains of delirium

A total of 14 delirium assessment tools addressed one to three delirium domains, each with a varying number of items, ranging from 1 to 58 criteria, based on observation and/or structured questions. The following tools addressed all three delirium domains—cognition, higher-level thinking, and circadian rhythm—as defined by Trzepacz et al. [35–40]: the DDT-Pro [40], the CAM-9 [46], the DOSS variants [98, 101], the DRS-R-98, the IAGe-D [28], the CAC-A [91], the CAC-B [96], and the Neecham [102]. In contrast, the RADAR, the VAS–AC [44, 100], and, the CAM-4 [43] the 4AT [40] did not cover all three delirium domains (Table 3).

**Table 3:** NH/LTC: content domains of delirium tools*

Delirium Tool	REF	Cognitive Domain^*^ (attention/vigilance plus other cognitive functions)	Higher Level Thinking Domain^*^ (language, thought process /comprehension)	Circadian Domain^*^ (motor activity, sleep–wake cycle)	Number of items meeting domain criteria n/N Items	% of domains addressed
		Items				
DDT-Pro	[40]	1	2	3	3/3	100%
CAM-4 S-CAM	[103] [104]	1, 2	3	-	3/4	66%
CAM-9	[5]	1, 2, 5, 6,7	3	8, 9	8/9	100%
DRS-R-98	[4]	2, 3, 4, 9,10, 11, 12. 13	5, 6,	1, 7, 8	13/13	100%
DOSS-13	[29]	2, 3, 4, 7, 8, 9, 12, 13	5	1, 6, 10, 11	13/13	100%
DOSS-25	[101]	4, 5, 6, 12, 13, 14, 22, 23, 24, 25	7, 8, 9, 10, 11	1, 2, 3, 15,16, 17, 18, 19,20,21	25/25	100%
I-AGeD	[28]	1, 2, 6	10	3, 4,5, 7, 8,9	9/10	100%
NEECHAM	[44]	Attention, Orientation	Verbal	Motor	4/7	100%
CAC-A	[44]	1,2,3, 19, 21	4, 14, 16	7, 13, 15, 17, 18	13/25	100%
CAC-B	[44]	3,4,5,27, 29,30, 31,32, 33,51,52	2,6,7,23, 48,56	1,11,15,19, 20,21,22, 37,38,40	28/58	100%
RADAR	[87]		2	1, 3	3/3	66%
4AT	[40]	2,3	-	1	3/4	66%
VAS–AC	[44]	-	1	-	1/1	33%

*4AT* 4 ‘A’s Test (Rapid Assessment Test for Delirium), *CAM-4* Confusion Assessment Method (4 Items), *CAM-9* Confusion Assessment Method (9 Items), *CAC-A* Cognitive Assessment of Confusion—Acute, *CAC-B* Cognitive Assessment of Confusion—Behavioral, *DDT-Pro* Delirium Diagnostic Tool—Provissional, *DOSS* Delirium Observation Screening Scale, *DOSS-13* Delirium Observation Screening Scale (13 Items), *DRS-R-98* Delirium Rating Scale-Revised-98, *NEECHAM* Neelon and Champagne Confusion Scale, *RADAR* Recognizing acute delirium as part of your routine, *S-CAM* Short Confusion Assessment Method, *VAS–AC* Visual Analog Scale for Acute Confusion

^*^The three delirium core domains were identified by a huge amount of research over several years by Trzepacz et al. [65–70]

### Validity and reliability of these tools

Comparing the sensitivity and specificity of the delirium tools we found that these properties were investigated in most of the tools. Against the reference standard DSM [1, 48–50], for the VAS–AC [44] and the CAC-A [44], the reported sensitivity was >90%, while a specificity >90% was reported for 4AT (cut off ≥4) [40] and the I-AGeD [28]. Using the CAM [43] as reference standard, sensitivity rates >90% were reported for DOSS 13 [29] and for the CAM, when research assistants were compared with bedside nurses, 75–100% [5, 61, 65].

The area under the curve (Receiver Operation Curve) values were reported for DRS-R-98 [4], DOSS-13 [29], 4AT [40] and DDT-pro [40] and CAM [65]; all the tools showed AUC–ROC >0.80. Based on these characteristics, the tool with the highest sensitivity and specificity with regard to the CAM (DSM-III-R) was the DOSS-13 [29] (Table 4).

**Table 4:** Psychometric properties of identified delirium tools in the context of studies in LTC/NH settings

Tool	Reference		Cronbach’s α	Cohen’s κ	ICC	Sensitivity	Specificity	Discriminant Validity	AUC–ROC	PPV	NPV	+LR	−LR	Reference Standard
CAC-A	Cacchione 2002	[44]	0.82			93.1	37.0	0.34° *p* < 0.01						DSM-IV criteria
CAC-B	Cacchione 2002	[44]	0.86	0.90		89.7	76.1	0.30° *p* < 0.01						DSM-IV criteria
VAS–AC	Cacchione 2002	[44]		0.80		96.6	80.5	0.21° *p* < 0.05						DSM-IV criteria
NEECHAM	Cacchione 2002	[44]	0.80	0.80		89.7	69.6	−0.30° *p* < 0.01						DSM-IV criteria
CAM-4	Voyer 2012	[103]				51.2	89.0			34.7	94.1			CAM
CAM-9*	Cole 2011	[5]		0.41–0.74		75–100	75–91							CAM
	Landreville 2013	[61]		0.53										Not reported due to Cohen’s K
	McCusker 2011a	[65]			0.90				0.82					CAM
S-CAM	Moon 2018	[104]		>0.99										S-CAM
	Teale 2018	[101]		0.80										CAM
DRS-R-98	Sepulveda 2015	[4]							>0.9					DSM-III-IV, -5 ICD-10
DOSS	Teale 2018 (cutoff ≥ 5)	[101]			0.71	61	71		0.66	1.3	99.5	2.1	0.55	CAM
DOSS-13	Sabbe 2024	[29]				97.1	95.4		0.97	70.8	96.7			CAM
4AT	Sepulveda 2021 (cutoff ≥ 4)	[40]				54.4	92.2		0.85	76.8	82.5	7.66	0.49	DSM-5
	Sepulveda 2021 (cutoff ≥ 3)	[40]				73.4	82.2							DSM-5
DDT-Pro	Sepulveda 2021 (cutoff ≤ 6)	[40				77.2	84.0		0.85	67.8	89.5	4.87	0.27	DSM-5
	Sepulveda 2021 (cutoff ≤ 7)	[40]				84.8	71.0							DSM-5
I-AGeD	Urfer 2022	[38]				0.60	0.94			0.38	0.97	9.60	0.43	DSM-5
RADAR	Voyer 2015	[87]				0.73	0.67			0.23	0.94			CAM

ICC, Intraclass Correlation Coefficient; *or not specified; °using the Geriatric Depression Scale

*4AT* 4 ‘A’s Test (Rapid Assessment Test for Delirium), *CAM-4* Confusion Assessment Method (4 Items), CAM-9* Confusion Assessment Method (9 Items), *CAC-A* Cognitive Assessment of Confusion—Acute, *CAC-B* Cognitive Assessment of Confusion—Behavioral, *DDT-Pro* Delirium Diagnostic Tool—Provissional, *DOSS* Delirium Observation Screening Scale, *DOSS-13* Delirium Observation Screening Scale (13 Items), *DRS-R-98* Delirium Rating Scale-Revised-98, *NEECHAM* Neelon and Champagne Confusion Scale, *RADAR* Recognizing acute delirium as part of your routine, *S-CAM* Short Confusion Assessment Method, *AS–AC* Visual Analog Scale for Acute Confusion

The inter-rater reliability when delirium was diagnosed on DSM or ICD criteria, ranged between 0.62 and 0.74 in Cohen’s Kappa (see Table 5).

**Table 5:** Inter-rater reliability of classifications systems

Tool	Reference		Cohen’s κ
DSM-III	Laurila 2003	[105]	0.74
DSM-III-R	Laurila 2003	[105]	0.74
	Sepulveda 2016	[67]	0.62
DSM-IV	Laurila 2003	[105]	0.72
	Sepulveda 2016	[67]	0.63
DSM-V	Sepulveda 2016	[67]	0.73
ICD	Laurila 2003	[105]	0.62

*DSM* diagnostic and statistical manual of mental disorders;

*ICD* International Classification of Diseases

## Discussion

With this scoping review we provide a comprehensive overview of delirium detection tools used in NH and LTC settings and their validity and reliability. We identified a total of 25 detection tools, including five variants of the CAM [43, 45, 47, 85, 92], two of the DOSS [97, 98], and six variants of the DSM [1, 48–50, 106] and ICD [88] classification systems, which were used as reference standard in delirium studies conducted in the NH/LTC setting. The most frequently used delirium tool was the CAM [43] in its long and short versions, as well as its adaptations [47], followed by the Neecham Confusion Scale [102]. However, of the 25 delirium tools, only 14 have undergone validity and reliability testing in the NH/LTC setting [4, 5, 28, 29, 40, 44, 61, 65, 101, 103, 104].

We examined which tools align to the three core domains of delirium, i.e., cognition, higher-level thinking, and circadian rhythm identified in phenomenological studies [40]. One tool stood out by achieving the highest concordance across all three domains while using the fewest items. This was the DDT-Pro [76] which used two items of the Cognitive Test for Delirium (CTD) entailing direct patient testing [107] and #3 from the DRS-R98 [90]. Over the last 54 years, the definition of delirium changed from organic brain syndrome (DSM-II) to delirium (DSM-III to DSM-5TR) [1, 48–50, 106].This partially influenced the development of delirium tools. The Brief Interview for Mental Status (BIMS) [85], the Nursing Home Confusion Assessment Method (NH-CAM) [45] and the Organic Brain Syndrome (OBS) scale [99] were neither defined nor validated on DSM criteria. The Clinical Assessment of Confusion-A (CAC-A) [91], the Clinical Assessment of Confusion-B (CAC-B) [96] and the Visual Analog Scale for Acute Confusion (VAS–AC) [100, 108] had non-DSM-based criteria, but were validated on DSM [44, 91, 96]. The Confusion Assessment Method (CAM) [43], the modified Confusion Assessment Method for the Emergency Department (mCAM-ED) [47], the Delirium Index (DI) [89], the Delirium Motoric Checklist [93], the Delirium-O-Meter (DOM) [86], the Delirium Rating Scale (DRS) [109] and its revision in 1998 (DRS-R-98) [90] as well as the Memorial Delirium Assessment Scale (MDAS) [95] were based and validated on DSM, but not validated in the LTC/NH setting. It should be noted that the timeframe during which the underlying definitions, i.e., DSM, evolved spans over 35 years. During this period, significant changes in definitions occurred. One particularly controversial change was the omission of the concept of disorganized thinking—a higher-level thinking function—from DSM-III-R to DSM-IV [37]. The justification for this change was that non-psychiatrists found it too difficult to identify. However, a substantial body of delirium research indicates the presence of disorganized thought processes in delirium [110]. This change is reflected in the development of the CAM [43] and the 4AT [111]: while the former includes disorganized thinking, aligning with DSM-III-R criteria, the latter reflects the omission of thought disorders from DSM-III-R to DSM-IV. Trzepacz and Meagher emphasized that the criterion of disorganized thinking is crucial for distinguishing between delirium and dementia [39, 57, 110]. This may explain why the 4AT [111] exhibited lower psychometric properties in Sepúlveda’s study in NH residents [40]. The same authors criticize the absence of disorganized thinking in the diagnostic criteria for delirium in DSM-5. It appears that the latest revision of the DSM criteria has sparked considerable criticism, particularly regarding the shift from “disturbance of consciousness” to “disturbance of attention” [112]. Our review identified a substantial but not perfect agreement (kappa ranging from 0.61 to 0.74) in the identification of residents with delirium using any DSM criterion. This indicates a considerable variability in the identification of delirium cases in this setting within the diagnostic framework considered as “reference standard”.

The best psychometric properties with regard to test accuracy, high sensitivity and specificity belonged to the 13-items-DOSS [29]. If a scale with the highest probability of a true positive result (+LR) is required, the I-AGeD [28] is the best choice. Demonstrating the best overall performance, both 4AT (cutoff ≥4) [40] and DDT-Pro (cutoff ≤6) [40] show strong results, with 4AT [40] exhibiting high specificity, a good AUC–ROC, and a strong positive likelihood ratio, while DDT-Pro [40] demonstrates high sensitivity and specificity, a strong AUC–ROC, and the lowest negative likelihood ratio, indicating a very reliable negative result.

Beyond the challenges posed by the psychometric properties of various delirium assessment tools, our review also identified variability among which professionals performed the delirium detection. Consequently, not every delirium tool is suitable for every profession. Furthermore, a distinction must be made between delirium monitoring and delirium assessment (episodic testing), as well as tools based on observation and/or structured questions. This has important implications for conducting structured assessments. For instance, in very old and frail populations, hearing loss is highly prevalent. As not all residents have hearing aids, the use of a hearing amplifier is essential when performing formal assessments, whether in clinical practice or clinical research. Thus, in delirium research utilizing structured assessments such as the 4AT [113], the omission of hearing amplifiers for participants with hearing impairment who do not use a hearing aid may introduce selection bias and/or measurement bias. Several national guidelines from the United Kingdom [114], the United States of America [51], Canada [115], Korea [23], and Switzerland [116] have been developed over time and can serve as a basis for implementing internal policies to prevent and manage delirium in long-term care facilities and nursing homes. A standardized and evidence-based approach to delirium detection and management, supported by appropriate training and the implementation of national guidelines, is essential to improving care quality and patient outcomes in NH and LTC settings. One key aspect to improving delirium detection in NH and LTC settings is targeted training for different professional groups. We propose that the level of training a professional receives correlates with the complexity of the tool they can effectively use. Physicians and advanced practice nurses may be better suited to apply comprehensive diagnostic criteria such as DSM-5 [1], whereas trained nurses could effectively utilize structured tools such as the DDT-Pro [40], the 4AT [40], or the CAM in its modern version, the mCAM-ED [47] for delirium detection. For delirium monitoring, the I-AGeD [28] demonstrates superior discriminatory ability compared to the DOSS [29], particularly in differentiating delirium from dementia. In addition, as the I-AGeD [28] was initially developed for laypersons, it can also be used by family members. Integrating a standardized combination of screening and diagnostic procedures into routine practice could enhance accuracy and early detection. A two-step approach, for instance, where a brief screening tool (e.g., DDT-Pro) [40] or I-Aged [28], is followed by a more detailed assessment of positive cases according to DSM-5 criteria, might be particularly effective. Future efforts should focus on the development of training programs that ensure all healthcare professionals involved in elderly care are proficient in using delirium detection tools, ultimately improving patient outcomes and reducing the burden of undetected delirium in NH/LTC settings.

## Strengths and limitations

With this scoping review we followed a rigorous methodology in the literature search, meeting the criteria for a systematic review. Although we did not search for grey literature, we are confident that we included all published interventional and observational studies reporting on delirium detection in NH and LTC settings. However, our findings do not necessarily mean that the tools most often reported in scientific literature are necessarily the same (or used in the same distribution) in daily practice within NH and LTC institutions. To gain a better understanding on what tools are used and by whom in these clinical settings, it would be necessary to conduct original research in representative national samples of NH and LTC institutions.

## Conclusions

With this scoping review we found evidence based on validity and reliability for 14 out of 25 delirium detection tools used in NH and LTC settings.

The DDT-Pro, the CAM-9, the DOSS variants, the DRS-R-98, the IAGe-D, the CAC-A, the CAC-B, and the Neecham reflect all three domains of delirium, i.e., the cognitive domain, the higher-level thinking domain, and circadian domain. The tool with the highest test accuracy was the 13-item-DOSS, followed by the DDT-Pro and the 4AT. As an alternative, the I-AGeD demonstrated promising findings in samples with a high percentage of people with dementia. Future research should focus on improving tool validation, integrating delirium screening and assessment into routine care, and training NH/LTC staff. Addressing these gaps could enhance early detection and management of delirium in this vulnerable population.

## Supplementary Information

Below is the link to the electronic supplementary material.

Supplementary file 1 (DOCX 27 KB)

Supplementary file 2 (DOCX 131 KB)

## Data Availability

No primary data were generated or analyzed in this study. All data used are from published sources, which are cited in the reference list.
