# Supplementary material for: Delirium in nursing homes and long-term care facilities: findings of a scoping review of detection tools
Source: Eur Geriatr Med. 2025 Jun 28;16(6):1919–31. doi: 10.1007/s41999-025-01250-8 (PMC12743713; doi:10.1007/s41999-025-01250-8)
Supplement: Supplementary file 1 — (DOCX 27 KB) [file 41999_2025_1250_MOESM1_ESM.docx]

**Supplementary Appendix S1.** Search strategy for different databases.

| **Database** | **Search Terms** | **Results** |
| --- | --- | --- |
| **PubMed-Medline** | ((((((((((((((((("Delirium"[Mesh] OR "Alcohol Withdrawal Delirium"[Mesh]) OR (delir*[Title/Abstract])) OR (confus*[Title/Abstract])) OR ("acute organic psychosyndrome"[Title/Abstract])) OR ("acute psycho-organic syndrome"[Title/Abstract])) OR ("psychomotor agitation"[Title/Abstract])) OR ("brain syndrome"[Title/Abstract])) OR ("delirium tremens"[Title/Abstract])) OR ("alcohol withdrawal delirium"[Title/Abstract])) OR ("transient cognitive dysfunction"[Title/Abstract])) OR ("mental disorientation"[Title/Abstract])) OR ("altered mental status"[Title/Abstract])) OR ("acute brain failure"[Title/Abstract])) OR ("acute cognitive impairment"[Title/Abstract])) OR (encephalopathy[Title/Abstract])) AND (((((((((((((((((("Nursing Homes"[Mesh]) OR "Long-Term Care"[Mesh]) OR ("nursing home*"[Title/Abstract])) OR ("long term care"[Title/Abstract])) OR ("residential care home*"[Title/Abstract])) OR ("long-term care"[Title/Abstract])) OR ("elderly care home*"[Title/Abstract])) OR ("assisted living facilit*"[Title/Abstract])) OR ("care home"[Title/Abstract])) OR ("skilled nursing facilit*"[Title/Abstract])) OR ("rest home"[Title/Abstract])) OR ("retirement home*"[Title/Abstract])) OR ("convalescent home*"[Title/Abstract])) OR ("senior care facilit*"[Title/Abstract])) OR ("adult care facilit*"[Title/Abstract])) OR ("aged care facilit*"[Title/Abstract])) OR ("retirement facilit*"[Title/Abstract])) OR ("old people* home*"[Title/Abstract]))) AND ((((((((((((((((("Psychometrics"[Mesh]) OR "Validation Studies as Topic"[Mesh]) OR ("psychometric*"[Title/Abstract])) OR ("validation"[Title/Abstract]) ) OR ("assess*"[Title/Abstract])) OR (diagnos*[Title/Abstract])) OR (screen*[Title/Abstract])) OR (identif*[Title/Abstract])) OR (evaluat*[Title/Abstract])) OR (test*[Title/Abstract])) OR (instrument*[Title/Abstract])) OR (exam*[Title/Abstract])) OR (scale*[Title/Abstract])) OR (tool*[Title/Abstract])) OR (risk*[Title/Abstract])) OR (prevent*[Title/Abstract])) OR (measure*[Title/Abstract]))) NOT (((((((((((("Hospitals"[Mesh]) OR "Intensive Care Units"[Mesh]) OR "Critical Care"[Mesh]) OR "Emergencies"[Mesh]) OR (hospital*[Title/Abstract]) ) OR (inhospital*[Title/Abstract])) OR (in-hospital*[Title/Abstract])) OR (operative*[Title/Abstract])) OR ("critical care"[Title/Abstract])) OR ("intensive care"[Title/Abstract])) OR (ICU[Title/Abstract])) OR (emergenc*[Title/Abstract])) | 476 |
| **Embase** |  | 25 |
| **CINAHL** | (((((MH "Delirium") OR (MH "Alcohol Withdrawal Delirium")) OR (AB delir*) OR (AB "confus*") OR (AB "acute organic psychosyndrome") OR (AB "acute psycho-organic syndrome") OR (AB "psychomotor agitation") OR (AB "brain syndrome") OR (AB "delirium tremens") OR (AB "alcohol withdrawal delirium") OR (AB "transient cognitive dysfunction") OR (AB "mental disorientation") OR (AB "altered mental status")) OR ((AB "acute brain failure") OR (AB "acute cognitive impairment") OR (AB encephalopathy))) AND (((MH "Nursing Homes") OR (MH "Long Term Care")) OR (AB "nursing home*") OR (AB ("residential care home*" OR "long term care facilit*" OR "elderly care home*" OR "assisted living facilit*" OR "care home*" OR "skilled nursing facilit*" OR "rest home*" OR "retirement home*" OR "convalescent home*" OR "senior care facilit*" OR "adult care facilit*" OR "aged care facilit*" OR "retirement facilit*" OR "old people* home*"))) AND (((MH "Psychometrics") OR (MH "Validation Studies")) OR (AB ("psychometric" OR "validation" OR "assess*" OR "detect*" OR "diagnos*" OR "screen* OR "identif*" OR "evaluat*" OR "test*" OR "instrument*" OR "exam*" OR "scale*" OR "tool*" OR "risk" OR "prevent*" OR "intervent*" OR "measure*)))) NOT ((MH "Hospitals") OR AB ("hospital*" OR "in-hospital" OR "inhospital" OR "operative*" OR "critical care" OR "intensive care" OR "ICU*" OR emergenc*)) | 186 |
| **PsycInfo** | (MJMAINSUBJECT.EXACT("Delirium") OR "delirium" OR MJMAINSUBJECT.EXACT("Organic Brain Syndromes") OR "acute brain syndrom*" OR "acute brain syndrome" OR "acute psycho-organic syndrome" OR "psychomotor agitation" OR "acute confusional state" OR MJMAINSUBJECT.EXACT("Mental Confusion") OR “mental confusion” OR MJMAINSUBJECT.EXACT("Organic Brain Syndromes") OR “organic brain syndrom*” OR “brain syndrome*” OR "toxic confusion" OR "delirium tremens" OR MJMAINSUBJECT.EXACT("Alcohol Withdrawal Syndrome") OR "Alcohol Withdrawal Syndrome" OR “transient cognitive dysfunction” OR “mental disorientation” OR “altered mental status” OR “acute brain failure” OR "acute cognitive impairment” OR MAINSUBJECT.EXACT("Toxic Encephalopathy") OR "Toxic Encephalopathy" OR Encephalopathy)  AND  (MJMAINSUBJECT.EXACT("Facilities") OR MJMAINSUBJECT.EXACT("Residential Care Institutions") OR MJMAINSUBJECT.EXACT("Treatment Facilities") OR MJMAINSUBJECT.EXACT("Nursing Homes") OR “nursing home*” OR MJMAINSUBJECT.EXACT("Health Care Services") OR MJMAINSUBJECT.EXACT("Long Term Care") OR MJMAINSUBJECT.EXACT("Residential Care Institutions") OR “Residential Care Institutions" OR “residential facilit*” OR “residential home*” OR "residential care" OR geriatric* OR “long-term care facility” OR “long term care facilit*” OR "elderly care home" OR "assisted living facility" OR "care home" OR “skilled nursing facility" OR "rest home" OR "retirement home" OR "convalescent home" OR "senior care facility" OR "adult care facility" OR "aged care facility" OR "retirement facility" OR "old people’s home")  AND  (MJMAINSUBJECT.EXACT("Diagnostic Criteria") OR “diagnostic criteria” OR psychometric OR tool* OR screen*OR instrument* OR scale* OR detect* OR diagnos* OR identif* OR check-list OR checklist OR "diagnostic evaluation" OR "screening tool" OR evaluation OR evaluation OR "diagnostic testing" OR identification OR "early detection" OR "diagnostic instrument" OR "delirium assessment" OR "delirium detection" OR "observational screening" OR "behavioral assessment" OR "cognitive function testing" OR "mental status examination" OR "cognitive impairment screening" OR "delirium screening scale" OR MJMAINSUBJECT.EXACT("Risk Assessment") OR "risk assessment" OR prevention OR "early prevention" OR "early identification" OR "early screening" OR "early intervention" OR "early diagnosis" OR "preventive measures" OR "risk reduction" OR "tool validation" OR validity OR "validation study" OR verification OR evaluation OR "construct validity" OR MJMAINSUBJECT.EXACT("Psychometrics") OR Psychometric*)  NOT  (MJMAINSUBJECT.EXACT.EXPLODE("Hospitals") OR hospital* OR inhospital OR in-hospital OR operative* OR perioperative* OR postoperative* OR "critical care" OR "intensive care unit" OR emergenc*) | 652 |
| **Cochrane database of systematic reviews** | (("delirium" OR "acute confus*" OR "acute organic psychosyndrome" OR confusion OR "brain syndrome" OR "delirium tremens" OR "alcohol withdrawal delirium" OR encephalopathy)  AND  ("nursing home*" OR "residential facilit*" OR "residential home*" OR "long-term care facility" OR "elderly care home" OR "senior care facility" OR "adult care facility")  AND  (tool* OR screen* OR instrument* OR scale* OR detect* OR diagnos* OR identif* OR "check-list" OR "diagnostic evaluation" OR "screening tool" OR evaluation OR "diagnostic testing" OR “identification” OR “assessment" OR “testing" OR "mental status examination" OR "risk assessment" OR “prevention” OR "preventive measures" OR "risk reduction" OR "tool validation" OR "validation study" OR verification OR evaluation OR "construct validity")  NOT  (hospital* OR "acute care" OR "in-hospital" OR ICU OR "emergency department" OR “acute care”)):ti,ab,kw | 166 |
| **Cochrane central register of controlled trials** | ('delirium'/exp OR 'delirium' OR 'confusion':ab,ti OR 'acute confusion':ab,ti OR 'delirium tremens':ab,ti OR 'alcoholic delirium':ab,ti OR 'brain disease':ab,ti)  AND  ('nursing home'/exp OR 'nursing home' OR 'residential home':ab,ti OR 'elderly care':ab,ti OR 'long term care':ab,ti OR 'assisted living facility':ab,ti OR 'rest home':ab,ti OR 'home for the aged':ab,ti)  AND  ('screening'/exp OR 'screening' OR 'tool':ab,ti OR 'assessment':ab,ti OR 'delirium tremens':ab,ti OR 'scale':ab,ti OR 'detect':ab,ti OR 'identity':ab,ti OR 'checklist':ab,ti OR 'diagnostic procedure':ab,ti OR 'evaluation study':ab,ti OR 'diagnostic test':ab,ti OR 'identification':ab,ti OR 'prevention':ab,ti OR 'risk reduction':ab,ti OR 'validation study':ab,ti)  NOT  ('hospital'/exp OR 'hospital' OR 'emergency care':ab,ti OR 'hospital patient':ab,ti OR 'intensive care':ab,ti OR 'emergency health service':ab,ti) | 277 |

Legend: CINAHL, Cumulative Index to Nursing and Allied Health Literature.
