# Supplementary material for: Delirium in nursing homes and long-term care facilities: findings of a scoping review of detection tools
Source: Eur Geriatr Med. 2025 Jun 28;16(6):1919–31. doi: 10.1007/s41999-025-01250-8 (PMC12743713; doi:10.1007/s41999-025-01250-8)
Supplement: Supplementary file 2 — (DOCX 131 KB) [file 41999_2025_1250_MOESM2_ESM.docx]

**Supplementary Appendix S2: Included Publications**

| **Study** | **1st author publication** | **Publi-cation year** | **Country** | **Study design** | **Setting** | **Population** | **Tool** | **HCP performing the screening/assessment** |
| --- | --- | --- | --- | --- | --- | --- | --- | --- |
| 1 | Andrew  [1] | 2006 | CA | Secondary analysis (Canadian Study of Health and Aging) | LTC Facilities and community-dwellers † | n=2,908  (mean age not reported) | - DSM-III-R criteria | - Nurse - Physician |
| 2 | Arinzon  [2] | 2011 | Israel | Observational prospective study | 3 Long-stay geriatric medical center, comprising LTC wards | n=322  mean age=79.8 | - CAM-9 - DRS | Not reported |
| 3 | Boockvar  [3] | 2013 | US | Observational prospective study | 3 NHs | n=136  mean age=76.2 | - CAM | Not reported |
| 4 | Boockvar  [4] | 2020 | US | RCT | 12 Units NH | n=219  mean age=81.7 | - CAM-4 - CAM-9 - CPS | - A single research assistant |
| 5 | Boorsma  [5] | 2012 | The Netherlands | Observational prospective study | NHs and residential care homes | n=2,193  (828 residents of nursing homes and 1365 residents of residential care homes)  (mean age not reported) | - NH-CAM | - Specially trained and supervised nursing assistants |
| 6 | Cacchione  [6] | 2002 | US | Observational prospective study | 2 LTC Facilities | n=74  mean age=82 | - CAC-A - CAC-B - NEECHAM - VAS-AC | - One investigator (PhD-prepared nurse) - One master’s-prepared gerontological nurse |
| 7 | Cacchione [7] | 2003b | US | Observational prospective study | 8 LTC setting | n=114  mean age=87.3 | - CAC-A - CAM - NEECHAM | - Trained study nurses |
| 8 | Ciampi  [8] | 2019 | CA | Observational prospective study | 7 LTC Facilities | n=276  mean age=77.2 | - CAM - DI | - Research assistant |
| 9 | Cole  [9] | 2011 | CA | Observational prospective study | 7 LTC Facilities | n=104  (mean age not reported) | - CAM-4 - DI | - Research staff (nurses and psychologists with experience in geriatrics) |
| 10 | Cole  [10] | 2013 | CA | secondary analysis of data collected for a prospective study | 7 LTC Facilities | n=273  (mean age not reported) | - CAM - DI | - Research assistant |
| 11 | Cole  [11] | 2017 | CA | Case–control study | 7 LTC Facilities | n=254  mean age=86.3 | - CAM | - Research assistant |
| 12 | Culp  [12] | 2003 | US | Quasi-experimental study | 7 LTC facilities | n=98  mean age=84.5 | - NEECHAM | - Research assistant (registered nurse) |
| 13 | Culp  [13] | 2008 | US | Observational prospective study | 13 LTC Facilities | n=312  mean age=86.1 | - CAM-4 - NEECHAM | - Registered nurse research assistants |
| 14 | DeCrane  [14] | 2011 | US | Observational prospective study | 13 LTC Facilities | n=320  (mean age not reported) | - CAC-A - CAM-4 - NEECHAM | Not reported |
| 15 | Dias  [15] | 2020 | Brasil | Cross-sectional study | 1 NH | n=135  mean age=85.7 | - CAM | - Nurse researcher |
| 16 | Dosa  [16] | 2007 | US | Observational retrospective study | NHs † | n=35,721  (mean age not reported) | - NH-CAM | Not reported |
| 17 | Fedecostante  [17] | 2024 | Italy | Cross-sectional study | 32 NHs | n=955  mean age=84.72 | - 4AT - DMSS | - Usual staff (physicians or nurses) |
| 18 | Franco  [18] | 2019 | Spain | Cross-sectional study | 1 SNF | n=200  (mean age not reported) | - DRS-R-98 - DSM-5 criteria | - Delirium research expert - Trained psychiatrist |
| 19 | Hadjistavropoulos  [19] | 2008 | CA | Cross-sectional study | 3 LTC Homes  3 LTC Units | n=152  (mean age not reported) | - CAM - DI | - Research nurses |
| 20 | Hölttä  [20] | 2014 | Finland | Observational prospective study | 7 acute geriatric wards  7 NHs  1 geriatric hospital | n=193  (mean age not reported) | - CAM - DSM-IV criteria | - Experienced geriatrician |
| 21 | Ishii  [21] | 2010 | US | Secondary analysis | 71 NHs | n=3,230  (mean age not reported) | - CAM | - Research nurses and NH nurses |
| 22 | Jones  [22] | 2010 | US | Analysis of screening data from a RCT | 8 NHs | n=4,744  (mean age not reported) | - CAM | - Trained non clinician interviewers |
| 23 | Jung  [23] | 2013 | US | Observational prospective study | 11.119 NHs | n=77,759  (mean age not reported) | - NH-CAM | Not reported |
| 24 | Kolanowski  [24] | 2013 | US | RCT | 8 SNFs | n=99  mean age=83.4 | - CAM-4 | - Trained research staff |
| 25 | Kosar  [25] | 2017 | US | Observational retrospective study | NH † | n=5,588,702  mean age=81.2 | - CAM-9 | - Nursing assessors |
| 26 | Lackner  [26] | 2008 | US | RCT | 12 SNFs | n=50  mean age=88.6 | - CAM | - Experienced research nurse practitioner |
| 27 | Landi  (data from the ULISSE project)  [27] | 2014 | Italy | Observational prospective study | 31 facilities (no more defined) | n=1,904  (mean age not reported) | - NH-CAM | - Trained staff (medical doctor and nurses) |
| 28 | Landreville  [28] | 2013 | CA | Cross-sectional study | 3 LTC facilities  1 LTC unit in a large regional hospital | n=155  (mean age not reported) | - CAM-9 | - Study nurses |
| 29 | Lapane  [29] | 2011 | US | RCT | 25 NHs | n=3,203  (mean age not reported) | - NH-CAM | - Nursing staff |
| 30 | Laurila  [30] | 2003 | Finland | Cross-sectional study | 2 geriatric hospitals  7 NHs | n=477 (195 in NH)  mean age=88.4 | - DSM-III criteria - DSM-III-R criteria - DSM-IV criteria - ICD-10 criteria | - Geriatricians - Nurses |
| 31 | Liu  [31] | 2023 | UK | Observational retrospective study | NH † | n=5,811  (mean age not reported) | - CAM - ICD-9/10 criteria | Not reported |
| 32 | Massimo  [32] | 2017 | US | Secondary analysis from RCT | 8 SNF | n=142  (mean age not reported) | - CAM | Not reported |
| 33 | Mayne  [33] | 2018 | Australia | Cross-sectional study | 5 NHs | n=450  (mean age not reported) | - CAM-9 | - Nursing staff |
| 34 | Mathillas  [34] | 2013 | Sweden and Finland | Cross-sectional study | Institutions  Home † | n=708  mean age=90.3 | - DSM-IV criteria - OBS scale | - Trained assessors (physicians, nurses, physical therapists or medical students) - Specialist in geriatrics |
| 35 | Mak  [35] | 2022 | US | Case series | 1 NH | n=40  mean age=82 | - CAM-4 | Not reported |
| 36 | Marcantonio  [36] | 2005 | US | Observational prospective study | 7 SNFs | n=504  (mean age not reported) | - CAM - DSI - MDAS | - Trained research assistant |
| 37 | Marcantonio  [37] | 2010 | US | RCT | 8 SNFs | n=457  (mean age not reported) | - CAM | - Research assistants |
| 38 | McCusker  [38] | 2011a | CA | Observational prospective study | 7 LTC Facilities | n=280  (mean age not reported) | - CAM-4 | - Research assistant (with support of primary nurse) |
| 39 | Mentes  [39] | 2003 | US | Quasi-experimental study | 4 LTC Facilities | n=49  (mean age not reported) | - NEECHAM | - Research staff |
| 40 | Moon  [40] | 2018 | Korea | Observational prospective study | 2 LTC Facilities | n=173  mean age=76.9 | - CAM-4 | - Staff nurses |
| 41 | Morichi  [41] | 2018 | Italy | Cross-sectional study | 71 NHs | n=1,454  mean age=84.4 | - 4AT - DMSS | - Physician |
| 42 | Pérez-Ros  [42] | 2019 | Spain | Cross-sectional study | 6 NHs | n=443  mean age=85.7 | - CAM - DSM-IV criteria | - Geriatrician |
| 43 | Pozzi  [43] | 2020 | Italy | Pre-post feasibility study | 1 NH | n=22  mean age=86.4 | - D-O-M - DSM-V criteria - m-RASS - RADAR | - Nurse - Expert geriatrician |
| 44 | Sabbe  [44] | 2024 | Belgium | Cross-sectional study | 6 LTC Facilities | n=338  (mean age) | - DOSS-13 - CAM-9 | Not reported |
| 45 | Saczynski  [45] | 2024 | US | Observational retrospective study | SNF † | n=740,838  (mean age not reported) | - CAM - ICD-9 | - Registered nurse or other staff |
| 46 | Santagata  [46] | 2021 | Italy | RCT | 2 NH | n=52  (mean age not reported) | - CAM | Not reported |
| 47 | Sepulveda  [47] | 2015 | Spain | Cross-sectional study | 1 SNF | n=125  mean age=78.7 | - DRS-98 - DSM IIIR - DSM-IV - DSM-V - ICD10 | - Trained researchers |
| 48 | Sepulveda [48] | 2016 | Spain | Observational prospective study | 1 SNF | n=200  (mean age not reported) | - DSM-III-R - DSM-IV - DSM-V - ICD10 | - Trained researchers |
| 49 | Sepúlveda  [49] | 2019 | Spain | Cross-sectional study | 1 NH | n=131  (mean age not reported) | - DSM-V criteria | - Psychiatrists - Resident in psychiatry - Psychologist |
| 50 | Sepúlveda  [50] | 2021 | Spain | Observational prospective study | 1 SNF | n=262  mean age=77.1 | - 4AT - DDT-Pro - DSM-V criteria | - Geriatrician |
| 51 | Siddiqi  [51] | 2016 | UK | Cluster randomized feasibility study | 14 care home | n=215  (mean age not reported) | - CAM - DRS-R-98 | - Trained research assistant |
| 52 | Skretteberg  [52] | 2022 | Norway | Observational prospective study | 3 NHs | n=145  mean age=84.2 | - CAM-4 | - Nurse staff - Physician |
| 53 | Teale  [53] | 2018 | UK | Observational prospective study | 9 NHs and residential care homes | n=216  mean age=84.9 | - DOSS-25 item | - Trained care home staff - Trained research staff |
| 54 | Urfer  [54] | 2022 | Switzerland | Observational prospective study | 1 NH | n=85  (mean age not reported) | - CAM-ED - I-AGeD | - Registered nurse - Advanced practice nurse |
| 55 | von Gunten  [55] | 2013 | Switzerland | Observational prospective study | 90 NHs | n=14,771  (mean age not reported) | - NH-CAM | Not reported |
| 56 | Voyer  [56] | 2012a | CA | Observational prospective study | 3 LTC Facilities | n=202  mean age=84.6 | - CAM-9 | - Nurses - Research assistant |
| 57 | Voyer  [57] | 2014 | CA | Observational prospective study | 7 LCT Facilities | n=280  (mean age not reported) | - CAM-9 - DSM-IV criteria | - Research assistant - Primary care nurses |
| 58 | Yang  [58] | 2009 | US | Observational prospective study | 8 Post Acute Care | n=441  mean age=84.1 | - DSI - MDAS | - Trained researcher |
| † no more details reported; 4AT = 4 'A's Test (Rapid Assessment Test for Delirium); CAB-B = Cognitive Assessment of Confusion – Behavioral; CAC-A = Cognitive Assessment of Confusion – Acute; CAM – S = Confusion Assessment Method – Severity; CAM = Confusion Assessment Method; DDT-pro = Delirium Diagnostic Tool – Professional; Delirium Index = Delirium Index (Score zur Bewertung des Deliriums); DMSS = Delirium Motor Subtype Scale; DOM = Delirium-O-Meter; DOSS = Delirium Observation Screening Scale; DRS-98 = Delirium Rating Scale-Revised-98; DSI = Delirium Symptom Interview; DSM III = Diagnostic and Statistical Manual of Mental Disorders = Third Edition; IAGe-D = Interdisciplinary Assessment of Geriatric Delirium; ICD = International Classification of Diseases; mCAM-ED = Modified Confusion Assessment Method for the Emergency Department; MDAS = Memorial Delirium Assessment Scale; NEECHAM = Neelon and Champagne Confusion Scale; NH CAM = Nursing Home Confusion Assessment Method; OBS scale = Organic Brain Syndrome Scale; RADAR = Recognizing Acute Delirium as Part of Your Routine; RASS modified = Richmond Agitation Sedation Scale (modified); VAS-AC = Visual Analog Scale for Acute Confusion | | | | | | | | |

**References**

1. Andrew MK, Freter SH, Rockwood K. Prevalence and outcomes of delirium in community and non-acute care settings in people without dementia: a report from the Canadian Study of Health and Aging. BMC Med. 2006;4:15. <https://doi.org/10.1186/1741-7015-4-15>.

2. Arinzon Z, Peisakh A, Schrire S, Berner YN. Delirium in long-term care setting: indicator to severe morbidity. Arch Gerontol Geriatr. 2011;52(3):270-5. <https://doi.org/10.1016/j.archger.2010.04.012>.

3. Boockvar K, Signor D, Ramaswamy R, Hung W. Delirium during acute illness in nursing home residents. J Am Med Dir Assoc. 2013;14(9):656-60. <https://doi.org/10.1016/j.jamda.2013.06.004>.

4. Boockvar KS, Judon KM, Eimicke JP, Teresi JA, Inouye SK. Hospital Elder Life Program in Long-Term Care (HELP-LTC): A Cluster Randomized Controlled Trial. J Am Geriatr Soc. 2020;68(10):2329-35. <https://doi.org/10.1111/jgs.16695>.

5. Boorsma M, Joling KJ, Frijters DH, Ribbe ME, Nijpels G, van Hout HP. The prevalence, incidence and risk factors for delirium in Dutch nursing homes and residential care homes. Int J Geriatr Psychiatry. 2012;27(7):709-15. <https://doi.org/10.1002/gps.2770>.

6. Cacchione PZ. Four acute confusion assessment instruments: reliability and validity for use in long-term care facilities. J Gerontol Nurs. 2002;28(1):12-9. <https://doi.org/10.3928/0098-9134-20020101-05>.

7. Cacchione PZ, Culp K, Dyck MJ, Laing J. Risk for acute confusion in sensory-impaired, rural, long-term-care elders. Clin Nurs Res. 2003;12(4):340-55. <https://doi.org/10.1177/1054773803253917>.

8. Ciampi A, Bai C, Dyachenko A, McCusker J, Cole MG, Belzile E. Latent class analysis of the multivariate Delirium Index in long-term care settings. Int Psychogeriatr. 2019;31(1):59-72. <https://doi.org/10.1017/S1041610218000510>.

9. Cole MG, McCusker J, Voyer P, Monette J, Champoux N, Ciampi A et al. Subsyndromal delirium in older long-term care residents: incidence, risk factors, and outcomes. J Am Geriatr Soc. 2011;59(10):1829-36. <https://doi.org/10.1111/j.1532-5415.2011.03595.x>.

10. Cole MG, McCusker J, Voyer P, Monette J, Champoux N, Ciampi A et al. Symptoms of delirium predict incident delirium in older long-term care residents. Int Psychogeriatr. 2013;25(6):887-94. <https://doi.org/10.1017/S1041610213000215>.

11. Cole MG, McCusker J, Wilchesky M, Voyer P, Monette J, Champoux N et al. Use of medications that antagonize mediators of inflammatory responses may reduce the risk of delirium in older adults: a nested case-control study. Int J Geriatr Psychiatry. 2017;32(2):208-13. <https://doi.org/10.1002/gps.4468>.

12. Culp K, Mentes J, Wakefield B. Hydration and acute confusion in long-term care residents. West J Nurs Res. 2003;25(3):251-66; discussion 67-73. <https://doi.org/10.1177/0193945902250409>.

13. Culp KR, Cacchione PZ. Nutritional status and delirium in long-term care elderly individuals. Appl Nurs Res. 2008;21(2):66-74. <https://doi.org/10.1016/j.apnr.2006.09.002>.

14. DeCrane SK, Culp KR, Wakefield B. Twelve-month mortality among delirium subtypes. Clin Nurs Res. 2011;20(4):404-21. <https://doi.org/10.1177/1054773811419497>.

15. Dias KM, Herdman TH, Ferretti-Rebustini REL, Lopes CT, Santos ERD. Relationships between nursing diagnoses and the level of dependence in activities of daily living of elderly residents. Einstein (Sao Paulo). 2020;18:eAO5445. <https://doi.org/10.31744/einstein_journal/2020AO5445>.

16. Dosa D, Intrator O, McNicoll L, Cang Y, Teno J. Preliminary derivation of a Nursing Home Confusion Assessment Method based on data from the Minimum Data Set. J Am Geriatr Soc. 2007;55(7):1099-105. <https://doi.org/10.1111/j.1532-5415.2007.01239.x>.

17. Fedecostante M, Balietti P, Di Santo SG, Zambon A, Marengoni A, Morandi A et al. Delirium in nursing home residents: is there a role of antidepressants? A cross sectional study. BMC Geriatr. 2024;24(1):767. <https://doi.org/10.1186/s12877-024-05360-z>.

18. Franco JG, Trzepacz PT, Gaviria AM, Sepulveda E, Vinuelas E, Palma J et al. Distinguishing characteristics of delirium in a skilled nursing facility in Spain: Influence of baseline cognitive status. Int J Geriatr Psychiatry. 2019;34(8):1217-25. <https://doi.org/10.1002/gps.5120>.

19. Hadjistavropoulos T, Voyer P, Sharpe D, Verreault R, Aubin M. Assessing pain in dementia patients with comorbid delirium and/or depression. Pain Manag Nurs. 2008;9(2):48-54. <https://doi.org/10.1016/j.pmn.2007.12.004>.

20. Holtta EH, Laurila JV, Laakkonen ML, Strandberg TE, Tilvis RS, Pitkala KH. Precipitating factors of delirium: stress response to multiple triggers among patients with and without dementia. Experimental gerontology. 2014;59:42-6. <https://doi.org/10.1016/j.exger.2014.04.014>.

21. Ishii S, Streim JE, Saliba D. Potentially reversible resident factors associated with rejection of care behaviors. J Am Geriatr Soc. 2010;58(9):1693-700. <https://doi.org/10.1111/j.1532-5415.2010.03020.x>.

22. Jones RN, Kiely DK, Marcantonio ER. Prevalence of delirium on admission to postacute care is associated with a higher number of nursing home deficiencies. J Am Med Dir Assoc. 2010;11(4):253-6. <https://doi.org/10.1016/j.jamda.2009.08.009>.

23. Jung HY, Meucci M, Unruh MA, Mor V, Dosa D. Antipsychotic use in nursing home residents admitted with hip fracture. J Am Geriatr Soc. 2013;61(1):101-6. <https://doi.org/10.1111/jgs.12043>.

24. Kolanowski A, Mulhall P, Yevchak A, Hill N, Fick D. The triple challenge of recruiting older adults with dementia and high medical acuity in skilled nursing facilities. Journal of nursing scholarship : an official publication of Sigma Theta Tau International Honor Society of Nursing / Sigma Theta Tau. 2013;45(4):397-404. <https://doi.org/10.1111/jnu.12042>.

25. Kosar CM, Thomas KS, Inouye SK, Mor V. Delirium During Postacute Nursing Home Admission and Risk for Adverse Outcomes. J Am Geriatr Soc. 2017;65(7):1470-5. <https://doi.org/10.1111/jgs.14823>.

26. Lackner TE, Wyman JF, McCarthy TC, Monigold M, Davey C. Randomized, placebo-controlled trial of the cognitive effect, safety, and tolerability of oral extended-release oxybutynin in cognitively impaired nursing home residents with urge urinary incontinence. J Am Geriatr Soc. 2008;56(5):862-70. <https://doi.org/10.1111/j.1532-5415.2008.01680.x>.

27. Landi F, Dell'Aquila G, Collamati A, Martone AM, Zuliani G, Gasperini B et al. Anticholinergic drug use and negative outcomes among the frail elderly population living in a nursing home. J Am Med Dir Assoc. 2014;15(11):825-9. <https://doi.org/10.1016/j.jamda.2014.08.002>.

28. Landreville P, Voyer P, Carmichael PH. Relationship between delirium and behavioral symptoms of dementia. Int Psychogeriatr. 2013;25(4):635-43. <https://doi.org/10.1017/S1041610212002232>.

29. Lapane KL, Hughes CM, Daiello LA, Cameron KA, Feinberg J. Effect of a pharmacist-led multicomponent intervention focusing on the medication monitoring phase to prevent potential adverse drug events in nursing homes. J Am Geriatr Soc. 2011;59(7):1238-45. <https://doi.org/10.1111/j.1532-5415.2011.03418.x>.

30. Laurila JV, Pitkala KH, Strandberg TE, Tilvis RS. The impact of different diagnostic criteria on prevalence rates for delirium. Dement Geriatr Cogn Disord. 2003;16(3):156-62. <https://doi.org/10.1159/000071004>.

31. Liu SH, Yuan Y, Baek J, Nunes AP, Pawasauskas J, Hume AL et al. Comparative safety of adding serotonin and norepinephrine reuptake inhibitors (SNRIs) versus nonsteroidal anti-inflammatory drugs (NSAIDs) to short-acting opioids for non-malignant pain in nursing homes. J Am Geriatr Soc. 2023;71(11):3390-402. <https://doi.org/10.1111/jgs.18519>.

32. Massimo L, Munoz E, Hill N, Mogle J, Mulhall P, McMillan CT et al. Genetic and environmental factors associated with delirium severity in older adults with dementia. Int J Geriatr Psychiatry. 2017;32(5):574-81. <https://doi.org/10.1002/gps.4496>.

33. Mayne S, Sundvall PD, Gunnarsson R. Confusion Strongly Associated with Antibiotic Prescribing Due to Suspected Urinary Tract Infections in Nursing Homes. J Am Geriatr Soc. 2018;66(2):274-81. <https://doi.org/10.1111/jgs.15179>.

34. Mathillas J, Olofsson B, Lovheim H, Gustafson Y. Thirty-day prevalence of delirium among very old people: a population-based study of very old people living at home and in institutions. Arch Gerontol Geriatr. 2013;57(3):298-304. <https://doi.org/10.1016/j.archger.2013.04.012>.

35. Mak W, Prempeh AA, Schmitt EM, Fong TG, Marcantonio ER, Inouye SK et al. Delirium after COVID-19 vaccination in nursing home residents: A case series. J Am Geriatr Soc. 2022;70(6):1648-51. <https://doi.org/10.1111/jgs.17814>.

36. Marcantonio ER, Kiely DK, Simon SE, John Orav E, Jones RN, Murphy KM et al. Outcomes of older people admitted to postacute facilities with delirium. J Am Geriatr Soc. 2005;53(6):963-9. <https://doi.org/10.1111/j.1532-5415.2005.53305.x>.

37. Marcantonio ER, Bergmann MA, Kiely DK, Orav EJ, Jones RN. Randomized trial of a delirium abatement program for postacute skilled nursing facilities. J Am Geriatr Soc. 2010;58(6):1019-26. <https://doi.org/10.1111/j.1532-5415.2010.02871.x>.

38. McCusker J, Cole MG, Voyer P, Monette J, Champoux N, Ciampi A et al. Prevalence and incidence of delirium in long-term care. Int J Geriatr Psychiatry. 2011;26(11):1152-61. <https://doi.org/10.1002/gps.2654>.

39. Mentes JC, Culp K. Reducing hydration-linked events in nursing home residents. Clin Nurs Res. 2003;12(3):210-25; discussion 26-8. <https://doi.org/10.1177/1054773803252996>.

40. Moon KJ, Park H. Outcomes of Patients With Delirium in Long-Term Care Facilities: A Prospective Cohort Study. J Gerontol Nurs. 2018;44(9):41-50. <https://doi.org/10.3928/00989134-20180808-08>.

41. Morichi V, Fedecostante M, Morandi A, Di Santo SG, Mazzone A, Mossello E et al. A Point Prevalence Study of Delirium in Italian Nursing Homes. Dement Geriatr Cogn Disord. 2018;46(1-2):27-41. <https://doi.org/10.1159/000490722>.

42. Perez-Ros P, Martinez-Arnau FM, Baixauli-Alacreu S, Caballero-Perez M, Garcia-Gollarte JF, Tarazona-Santabalbina F. Delirium Predisposing and Triggering Factors in Nursing Home Residents: A Cohort Trial-Nested Case-Control Study. J Alzheimers Dis. 2019;70(4):1113-22. <https://doi.org/10.3233/JAD-190391>.

43. Pozzi C, Lanzoni A, Lucchi E, Salimbeni I, DeVreese LP, Bellelli G et al. Activity-based occupational therapy intervention for delirium superimposed on dementia in nursing home setting: a feasibility study. Aging Clin Exp Res. 2020;32(5):827-33. <https://doi.org/10.1007/s40520-019-01422-0>.

44. Sabbe K, van der Mast R, Dilles T, Van Rompaey B. Validation of the Delirium Observation Screening Scale in long-term care facilities in Flanders. Geriatr Gerontol Int. 2024;24(6):619-25. <https://doi.org/10.1111/ggi.14878>.

45. Saczynski JS, Koethe B, Fick DM, Vo QT, Devlin JW, Marcantonio ER et al. Cognitive and functional change in skilled nursing facilities: Differences by delirium and Alzheimer's disease and related dementias. J Am Geriatr Soc. 2024;72(11):3501-9. <https://doi.org/10.1111/jgs.19112>.

46. Santagata F, Massaia M, D'Amelio P. The doll therapy as a first line treatment for behavioral and psychologic symptoms of dementia in nursing homes residents: a randomized, controlled study. BMC Geriatr. 2021;21(1):545. <https://doi.org/10.1186/s12877-021-02496-0>.

47. Sepulveda E, Franco JG, Trzepacz PT, Gaviria AM, Vinuelas E, Palma J et al. Performance of the Delirium Rating Scale-Revised-98 Against Different Delirium Diagnostic Criteria in a Population With a High Prevalence of Dementia. Psychosomatics. 2015;56(5):530-41. <https://doi.org/10.1016/j.psym.2015.03.005>.

48. Sepulveda E, Franco JG, Trzepacz PT, Gaviria AM, Meagher DJ, Palma J et al. Delirium diagnosis defined by cluster analysis of symptoms versus diagnosis by DSM and ICD criteria: diagnostic accuracy study. BMC psychiatry. 2016;16:167. <https://doi.org/10.1186/s12888-016-0878-6>.

49. Sepúlveda E, Franco JG, Leunda A, Moreno L, Grau I, Vilella E. Delirium clinical correlates and underdiagnosis in a skilled nursing home. The European Journal of Psychiatry. 2019;33(4):152-8. <https://doi.org/10.1016/j.ejpsy.2019.06.001>.

50. Sepulveda E, Bermudez E, Gonzalez D, Cotino P, Vinuelas E, Palma J et al. Validation of the Delirium Diagnostic Tool-Provisional (DDT-Pro) in a skilled nursing facility and comparison to the 4 'A's test (4AT). Gen Hosp Psychiatry. 2021;70:116-23. <https://doi.org/10.1016/j.genhosppsych.2021.03.010>.

51. Siddiqi N, Cheater F, Collinson M, Farrin A, Forster A, George D et al. The PiTSTOP study: a feasibility cluster randomized trial of delirium prevention in care homes for older people. Age and ageing. 2016;45(5):652-61. <https://doi.org/10.1093/ageing/afw091>.

52. Skretteberg WH, Holmefoss I, Krogseth M. Delirium During Acute Events in Nursing Home Patients. J Am Med Dir Assoc. 2022;23(1):146-9. <https://doi.org/10.1016/j.jamda.2021.10.006>.

53. Teale EA, Munyombwe T, Schuurmans M, Siddiqi N, Young J. A prospective observational study to investigate utility of the Delirium Observational Screening Scale (DOSS) to detect delirium in care home residents. Age and ageing. 2018;47(1):56-61. <https://doi.org/10.1093/ageing/afx155>.

54. Urfer Dettwiler P, Zuniga F, Bachnick S, Gehri B, de Jonghe JFM, Hasemann W. Detecting delirium in nursing home residents using the Informant Assessment of Geriatric Delirium (I-AGeD): a validation pilot study. Eur Geriatr Med. 2022;13(4):917-31. <https://doi.org/10.1007/s41999-022-00612-w>.

55. von Gunten A, Mosimann UP, Antonietti JP. A longitudinal study on delirium in nursing homes. Am J Geriatr Psychiatry. 2013;21(10):963-72. <https://doi.org/10.1016/j.jagp.2013.01.003>.

56. Voyer P, McCusker J, Cole MG, Monette J, Champoux N, Ciampi A et al. Prodrome of delirium among long-term care residents: what clinical changes can be observed in the two weeks preceding a full-blown episode of delirium? Int Psychogeriatr. 2012;24(11):1855-64. <https://doi.org/10.1017/S1041610212000920>.

57. Voyer P, McCusker J, Cole MG, Monette J, Champoux N, Ciampi A et al. Nursing documentation in long-term care settings: New empirical evidence demands changes be made. Clin Nurs Res. 2014;23(4):442-61. <https://doi.org/10.1177/1054773813475809>.

58. Yang FM, Marcantonio ER, Inouye SK, Kiely DK, Rudolph JL, Fearing MA et al. Phenomenological subtypes of delirium in older persons: patterns, prevalence, and prognosis. Psychosomatics. 2009;50(3):248-54. <https://doi.org/10.1176/appi.psy.50.3.248>.
